# Immunoglobulin subclass in experimental murine *Toxocara cati* infection

**DOI:** 10.14202/vetworld.2017.1333-1336

**Published:** 2017-11-13

**Authors:** Kusnoto, Setiawan Koesdarto, Sri Mumpuni, R. Heru Prasetyo

**Affiliations:** 1 Department of Parasitology, Faculty of Veterinary Medicine, Universitas Airlangga, Surabaya, East Java, Indonesia; 2 Department of Parasitology, Faculty of Medicine, Universitas Airlangga, Surabaya, East Java, Indonesia

**Keywords:** diagnostic kit, enzyme-linked immunosorbent assay, human, immunoglobulin G subclass, monoclonal antibody, toxocariasis, *Toxocara cati*

## Abstract

**Aim:**

The aim of this study was to detect specific immunoglobulin (Ig) that could be used to determine monoclonal antibody in conjugate-making an effort for the indirect enzyme-linked immunosorbent assay (ELISA) diagnostic kit of toxocariasis in human.

**Materials and Methods:**

The study was conducted to assess the Ig profile, based on ELISA-isotyping, in mice infected with second stage larvae eggs of *Toxocara cati*. The optical density values of anti-*T. cati* mice serum IgG subclasses were analyzed by applying ANOVA factorial.

**Results:**

The specific IgG subclass in mice infected with *T. cati* mice was found to be IgG2β.

**Conclusion:**

Subclass of IgG, especially IgG2β, can provide leads about the use of the monoclonal antibody in conjugate making an effort for the indirect ELISA diagnostic kit.

## Introduction

The disease caused by *Toxocara* *cati* infection in humans is a helminth zoonosis, which is mostly found in children [1,2]. However, there were some cases recorded in adults also [3]. Toxocariasis, caused by *T. cati* needs more attention due to the high population of cats in Indonesia and the close association of these pets to humans. The habit of a cat to hide its feces after defecation may prolong the survivability of worm eggs in the soil [4]. Furthermore, we should also be aware of a high prevalence of toxocariasis in Surabaya. It was reported that the prevalence of toxocariasis in stray cats was about 60.9% of 69 samples [5]. About 31.9% of soil around slaughterhouses and about 20.6% of the soil around dairy farms were reported to contain eggs of *Toxocara* spp. included *T. cati* [6]. This situation could increase the risk of toxocariasis [7]. This finding supports the fact that human toxocariasis from infective eggs, containing second-stage (L2) larvae [8]. These infections were called human toxocariasis, referring to one of most common helminth zoonosis [9]. Human toxocariasis was the most prominent of all nematode diseases because it caused major health problems in children and ocular destruction on adults. Human toxocariasis has been classified into two categories, visceral toxocariasis and ocular toxocariasis due to visceral larvae migrans and ocular larvae migrans [10]. These had been global concern that *T. cati*, including visceral and ocular larvae migrans as their clinical manifestations were the main source of infections in human [11]. Considering the fatality due to ocular or cerebral larvae migrans, immunodiagnosis in human should be conducted. The presence of larvae in body tissues would trigger immune responses marked by IgE and an increase in the count of eosinophils leading to a specific type of antibody-dependent cell mediated cytotoxicity [12].

Immunological diagnosis of human toxocariasis conducted through suspected blood serum examination required high sensitivity and specificity. Enzyme-linked immunosorbent assay (ELISA) technique is reliable for its high sensitivity. In indirect ELISA technique, in addition to needing *T. cati* antigens and suspect animal serum containing polyclonal antibody, it also requires enzyme-labeled monoclonal antibodies (conjugate) as biomarkers [13].

The aim of this study was to detect specific immunoglobulin (Ig) that could be used to determine monoclonal antibody in conjugate-making effort for the indirect ELISA diagnostic kit of human toxocariasis.

## Materials and Methods

### Ethical approval

The present study was approved by Ethical Committee vide Ethical Clearance No. 285-KE Animal Care and Use Committee, Faculty of Veterinary Medicine, Universitas Airlangga.

### Research animals

In this research, feral cats were used as source of worms which were caught from several traditional markets in Surabaya. Male mice *Balb/c*, 8 weeks age was used to examine humoral immune response.

### Collecting adult *T. cati*. and culturing worm eggs to obtain second-stage *T. cati* larvae (L2)

The feral cats were quarantined and underwent fecal examination to find out the presence of *T. cati* eggs. The cats without toxocariasis were released, while five cats with *T. cati* were administered piperazine adipate as anthelmintic at 50 mg/kg body weight with the consideration that those cats small and thin and not causing the worm to die and observed for 1 to 3 days to obtain adult worms which were excreted in feces [13,14].

*T. cati* worms collected from cats were placed in Erlenmeyer flasks containing warm (37°C) phosphate buffered saline (PBS) (pH 7.4). About 50 ml PBS was added to every 20 worms and incubated in a water bath at a 37°C temperature for 15-20 min. PBS solution was replaced, and incubation continued for another 15 min to an hour. All living worms were transferred to fresh PBS solution and incubated again for 4 h. The live worms were collected, and the residual solutions were filtered using plastic T200 filters (which equals to 25 µm). The residue contained *T. cati*. eggs [15-17].

The eggs were cultured in PBS medium at room temperature for 21-28 days until second-stage larvated (L2) eggs.

### Infection of mice with second-stage of *T. cati* larvated EGGS

Six mice were infected *per os* by second-stage *T. cati* larvated eggs (L2). On the 0, 7^th^, and 14^th^ days after infection, sera samples were collected to examine Ig subclasses: IgG1, IgG2, IgG2, and IgG3 by applying ELISA sub-isotyping kit technique.

*T. cati* antigen (2 µg/l) diluted with carbonate buffer (50 mmol/l carbonate, pH 9.6) then adsorbed on ELISA microplate 100 µl each well and incubated at 40°C for one night. The microplate is then blocked with a blocking buffer (1% BSA, 0.02% NaN_3_ in PBS) and incubated at 37°C for 1 h. Then washed with a washing buffer (0.15 M NaCl, 0.05% Triton X-100, 0.02% NaN_3_) 3 times. Tested antibodies were inserted into each well (100 µl) and incubated at 37°C for 1 h, then washed 3 times with washing buffer, followed by addition of an antisubclass of IgG (Mouse Typer Sub-Isotyping Kit, Bio-rad) diluted with blocking buffer (1:1000 dilution) of 100 µl each well and incubated at 37°C for 1 h. Then microplate washed again with washing buffer and added conjugate (rabbit anti-mouse IgG labeled with alkaline phosphatase enzyme) diluted with blocking buffer (1:1000 dilution) 100 µl each well and incubated at 37°C for 1 h. The microplate was washed again with washing buffer for subtract added 100 µl each well and incubated for 10-30 min in dark space. Recharge is then read with ELISA reader at 405 nm [13].

### Statistical analysis

The optical density values of anti-*T. cati* mice serum IgG subclasses were analyzed by applying analysis of variance factorial.

## Results and Discussion

The optical density values of anti-*T. cati* mice serum IgG subclasses observed at different time periods indicated different results (p<0.01). The highest optical density values (0.384±0.199) were found on the 28^th^ day indicating a significant difference compared to other observations. The second and third highest average optical density values were found on the 7^th^ (0.279±0.099) and 14^th^ day (0.275±0.119) observations. These two values were not significantly different (p>0.05), but these values were significantly different from the 0 to 28^th^ day observations.

Average optical density values of anti-*T. cati* mice sera for Ig-G1, Ig-G2α, Ig-G2β, and Ig-G3 were 0.227, 0.342, 0.374, and 0.170, respectively. These values showed a highly significant difference (p<0.01) among subclass Ig. Regardless of the observation time, the result of HSD 5% examination indicated that the highest optical density value was found in Ig-G2β. This value was not significantly different from Ig-G2α but it was significantly different from Ig-G1 to Ig-G3.

The statistical analysis results using ANOVA factorial on optical density of anti-*T. cati* mice sera indicated an interaction between observation time and IgG subclasses found. Average notations and standard deviations of combined treatments were presented in Table-1. The highest optical density average values were found on Ig-G2β subclass on the 28^th^ day observation. The same results (p>0.05) were found on combined treatments of Ig-G2α subclass on the 28^th^ day observation, Ig-G2β subclass on the 14^th^ day observation, and Ig-G2α subclass on the7^th^ day observation. Meanwhile, the lowest optical density average values were found on Ig-G3 subclass indicating a nonsignificant difference (p>0.05) with Ig-G1, Ig-G2α, and Ig-G2β subclasses on the 0 day observation and Ig-G1 and Ig-G3 subclasses on the 7^th^ and 14^th^ observation days.

**Table-1:** Optical density values of anti-*T. cati* mice sera of treatment groups based on time and IgG subclasses combination.

Time	IgG subclasses	Average±SD
0 day PI	Ig-G1	0.168^ab^±0.019
	Ig-G2α	0.232^abcd^±0.046
	Ig-G2β	0.190^abc^±0.052
	Ig-G3	0.110^a^±0.013
7 days PI	Ig-G1	0.230^abcd^±0.038
	Ig-G2α	0.314^bcd^±0.053
	Ig-G2β	0.365^cde^±0.072
	Ig-G3	0.207^abc^±0.128
14 days PI	Ig-G1	0.243^abcd^±0.085
	Ig-G2α	0.306^bcd^±0.115
	Ig-G2β	0.400^de^±0.072
	Ig-G3	0.153^ab^±0.029
28 days PI	Ig-G1	0.267^abcd^±0.129
	Ig-G2α	0.516^e^±0.222
	Ig-G2β	0.540^e^±0.115
	Ig-G3	0.212^abcd^±0.022

a,b,c,d,e Values in the same column with different superscripts indicate significant difference at p<0.01 (n=6). *T. cati=Toxocara cati,* IgG=Immunoglobulin G, SD=Standard deviation

Based on the observation time, the highest anti-*T. cati* mice blood sera optical density value was found on the 28^th^ observation day indicating a significant difference (p<0.05) compared to other observation times, followed by results on the 7^th^ and 14^th^ observation days. Meanwhile, the lowest optical density value was found on day 0. When compared with other studies which use anti-*Toxocara canis* mice sera, the highest optical density value was also found on the 28^th^ observation day while the lowest one was also found on day 0 observation indicating nonsignificant difference (p>0.05) with the 7^th^ and 14^th^ observation days. If this finding confirmed with the other experiment using rabbit specific anti *T. canis* IgG and excretory-secretory antigen applying ELISA technique to diagnose toxocariasis, positive responses could be detected at the 20^th^ day after inoculation [18]. It conducted artificial infection proposed that rabbit immune response tended to increase until 60^th^ day and stabilized until 210^th^ day after inoculation. Antibody titers could be detected for the first time at 15^th^ day after inoculation and rapid increase was found at 28^th^ to 58^th^ day after inoculation [19].

The highest average optical density value of mice blood sera infected by L2 of *T. cati* regardless observation time was found with Ig-G2β which indicates nonsignificant difference from Ig-G2α (p>0.05). The lowest optical density values were found on Ig-G1 and Ig-G3 (Table-1).

It was found out that the highest antibody response at 7^th^ day after infection. The second peak was found when L2 hatched and during larvae migration to visceral organs. Immune response on stimulated all Ig classes [12]. However, it seemed that IgG2 was more prominent compared to other Ig during *T. cati* infection. IgG2 production was stimulated by interferon-gamma secreted by CD8^+^Th1 [20].

## Conclusion

Based on the findings of this study, it can be concluded that the most dominant (specific) Ig subclass found on L2-*T. cati* infected mice blood sera was Ig-G2β, and it can provide leads about the use of the monoclonal antibody in conjugate-making effort for the indirect ELISA diagnostic kit.

## Authors’ Contributions

K: Research coordinator, prepared antigen and antibody, and statistical analysis. SK: Method of Indirect ELISA and ELISA-Isotyping. SM: Prepared mice, and sampling blood mice. RHP: Drafted and revised the manuscript and corresponding author. All authors read and approved the final manuscript.
